# **Additional morphological data and first genetic characterization on females of** ***Micropleura vazi*** **Travassos, 1933, parasitic in** ***Caiman yacare*** **from Brazil, with comments on the phylogeny of Dracunculoidea (Nematoda)**

**DOI:** 10.1007/s11230-026-10291-x

**Published:** 2026-07-26

**Authors:** Glória M. C. Lacerda, Fernando Paiva, Luiz E. R. Tavares, João A. de Araujo Filho, Samuel C. Ribeiro, Felipe B. Pereira

**Affiliations:** 1 Programa de Pós-Graduação em Parasitologia, Universidade Federal de Minas Gerais, Av. Antônio Carlos 6627, Pampulha, Belo Horizonte, MG CEP 31270-901 Brazil; 2 Instituto de Biociências, Universidade Federal de Mato Grosso do Sul, Av. Costa e Silva s/n, Campo Grande, MS CEP 99070-900 Brazil; 3 Centro de Ciências Biológicas e da Saúde, Departamento de Ciências Biológicas, Universidade Regional do Cariri, Rua Vicente Alexandrino de Alencar 348, Centro, Campos Sales, CE CEP 63150-000 Brazil; 4 Laboratório de Biologia e Ecologia de Animais Silvestres, Instituto de Formação de Educadores, Universidade Federal do Cariri, Rua Olegário Emidio de Araujo s/n, Brejo Santo, CE CEP 63260-000 Brazil

## Abstract

Species of *Micropleura* Linstow, 1906 (Dracunculoidea: Nematoda) are common parasites of crocodilians worldwide. Studies related to this genus remain scarce, particularly regarding genetic characterizations, due to the difficulty in obtaining the parasite specimens. Although the morphology of *M*. *vazi* Travassos, 1933, a parasite of *Caiman* spp. in the Neotropics, has been previously evaluated, some morphological inconsistencies persist, and certain features are insufficiently described. Moreover, there is no genetic data for the species. In this regard, we provide the first genetic characterization of *M*. *vazi* along with detailed morphological analysis using light and scanning electron microscopy (SEM). We report for the first time in *M*. *vazi* the presence of phasmids and of oesophageal gland nuclei, in addition to provide a clearer view of the cephalic region and the accurate position of the vulva. Genetic characterization of the species was conducted using partial sequences of the 18S and 28S rDNA, and the phylogenetic reconstructions using these first indicated the monophyly of *Micropleura* and of most families within Dracunculoidea. Moreover, Micropleuridae appears to be the sister group to Dracunculidae, and Philonematidae should be considered valid as it is not close to Philometridae. Furthermore, Anguillicolidae should be allocated in another superfamily, Anguillicoloidea, which is basal relative to Dracunculoidea and far from it. The absence of males in the current sampling may suggest a seasonal occurrence of these parasites, as has been reported for other dracunculoid nematodes.

## Introduction

The genus *Micropleura* Linstow, 1906 was established by Linstow (1906) with *M. vivipara* Linstow, 1906 as the type species, a parasite of the crocodile *Gavialis gangeticus* (Gmelin) from India. However, in the original description the genus was only assigned to the Nematoda (Linstow, 1906). Therefore, Baylis and Daubney (1922) proposed the subfamily Micropleurinae within the family Filariidae, to accommodate this genus. Travassos (1933a), in a review on parasites of South American crocodiles, later reallocated *Micropleura* to the family Philometridae. The current accepted classification places *Micropleura* in the family Micropleuridae (Baylis & Daubney, 1922) and superfamily Dracunculoidea (Anderson et al., 2009). Regarding its diagnostic aspects, *Micropleura* is characterized by a rounded and prominent anterior end, six small papillae arranged in a circle, a small, circular oral opening, absence of labia and teeth at the anterior end, and a rounded caudal end in both males and females (Linstow, 1906; Travassos, 1933a; Anderson et al., 2009).

*Micropleura* species have been recorded from all crocodilian families, including Gavialidae, Crocodylidae, and Alligatoridae, in addition to some trionychid (Trionychidae) chelonians (Junker & Mutafchiev, 2017). Currently, five congeners are recognized valid: *M. australiensis* Moravec, Kay & Hobbs, 2004, a parasite of *Crocodylus johnsoni* Krefft (type host) and *Cr. porosus* Schneider (Crocodylidae) in Australia; *M. indica* Khera,1951 (syn. *M*. *trionyxi* and *M*. *lissemysia*), found in *Nilssonia gangetica* (Cuvier) (syn. *Trionyx gangeticus*) (type host) and *Lissemys punctata* (Trionychidae) in India (see Moravec et al., 2004 for synonymy); *M. vazi* Travassos, 1933 of *Caiman crocodilus* (Linnaeus) (syn. *Caiman sclerops*) (Alligatoridae) in Brazil and Venezuela; *M. vivipara* Linstow, 1906 infecting *G. gangeticus* in India; and *M. huchzermeyeri* Junker & Mutafchiev, 2017 found in *Cr*. *niloticus* Laurenti, in South Africa (Travassos, 1933a, b; Khera, 1951; Siddiqi & Jairajpuri, 1963; Moravec & Prouza, 2003; Moravec et al., 2004; Junker & Mutafchiev, 2017).

Species of the genus *Caiman* are distributed throughout Central and South America (Vitt & Caldwell, 2014; Amavet et al., 2023), and may harbour diverse communities of parasites, including trematodes, nematodes, and acanthocephalans (Catto & Amato, 1993; Werneck & Leandro, 2021). *Caiman yacare* is one of three species of the genus present in Brazil (Guedes et al., 2023) and is among the most extensively studied in terms of conservation. However, limited research has addressed its parasitism (Gorza et al., 2025).

According to Moravec et al. (2004), the morphology from most of the *Micropleura* spp. is poorly known. In fact, most congeners have not been described in a consist way, and certain variations in important taxonomic features have been reported (Junker & Mutafchiev, 2017). Moreover, genetic characterization is currently available only for *M*. *australiensis*. In this sense, the present study aimed to provide detailed morphological data and genetic characterization of female *M. vazi* specimens*,* collected from the body cavity of *C*. *yacare* in the Pantanal wetlands, Brazil, using light and scanning electron microscopy (SEM) for enhanced morphological observations.

## Material and methods

### Collection, processing and morphological evaluation of parasites

On 18 August 2018 one specimen of *C. yacare* (approximately 1.5 m in total length) was found freshly dead by researchers of the Universidade Federal de Mato Grosso do Sul, at a marginal lagoon of the federal highway BR 262, which crosses the Pantanal wetlands, municipality of Corumbá, State of Mato Grosso do Sul, Brazil. Host nomenclature and classification follow Uetz et al. (2026). The reptile was necropsied immediately in the field, and the nematodes were found alive within the body cavity. These parasites were washed in saline, fixed in hot 4% formalin and preserved in 70% ethanol. The middle body part of one specimen was excised and fixed in molecular-grade 96–99% ethanol for genetic studies; the remaining parts (anterior and posterior) were processed for morphological evaluation as previously described.

For light microscopy, specimens were cleared in glycerine and observed in a Nikon Eclipse Ei (Nikon Corporation, Tokyo, Japan), microscope equipped with a PrimeCam Intervision 12 (Prime Life Science, Boca Raton, FL, USA) attached, which was used for measurements and image capture. Drawings were made in an Olympus CH2 (Olympus, Tokyo, Japan) microscope with a drawing tube attached. For scanning electron microscopy (SEM) two females were dehydrated through a graded ethanol series, dried by evaporation with hexamethyl disilazane and sputter-coated with gold (10 nm layer). The coated specimens were then observed in a JEOL JSM 6460-LV SEM (JEOL, Tokyo, Japan), at an accelerating voltage of 15 kV. Measurements are presented as ranges and in micrometres, unless indicated otherwise. Voucher specimens were deposited in the Coleção Helmintológica do Instituto Oswaldo Cruz (CHIOC).

### Genetic procedures

Genomic DNA was isolated using DNeasy Blood & Tissue Kit (QIAGEN, Hilden, Germany), following the manufacturer’s instructions. Regions of the nuclear 18S and 28S rDNA were amplified, using the set of primers Nema18SF + Nema18SR (Floyd et al., 2005) and D2A + D3B (Nunn, 1992). The polymerase chain reactions (PCR) and cycling conditions were the same as those used by Ailán-Choke et al. (2023). An enzymatic treatment with ExoSAP-IT (ThermoFisher, Waltham, MA, USA) was made to purify PCR products that were sent for sequencing at Instituto René Rachou (Fiocruz Minas, Belo Horizonte, Brazil). Contiguous sequences were assembled and inspected, primers were trimmed, and the consensus was extracted in Geneious Prime (Dotmatics, Boston, MA, USA) and deposited in GenBank. A preliminary BLAST search on the GenBank database (https://www.ncbi.nlm.nih.gov/Blast.cgi) was performed to confirm the genetic proximity between the present samples and those from other species of *Micropleura*, as well as other dracunculoid nematodes.

### Phylogenetic analysis

Due to the data available for the only congener (*M*. *australiensis*) that has been genetically characterized, as well as for representatives from different families of Dracunculoidea, the phylogenetic reconstruction was based on 18S sequences. Our main phylogenetic objectives were to evaluate the monophyly of *Micropleura* and, consequently, of Micropleuridae, and to evaluate the monophyly of other families within the Dracunculoidea. *Camallanus lacustris* (Zoega, 1776) was chosen as outgroup base on previous phylogenies including dracunculoid nematodes (Wijová et al., 2006; Černotíková et al., 2011).

The sequences used for phylogenetic analysis and their respective details, are listed in Table 1. Sequences were aligned using M-Coffee (Notredame et al., 2000) and evaluated by the transitive consistency score, to verify the reliability of aligned positions, trim the sites with low scores and improve the phylogenetic resolution (Chang et al., 2014).

**Table 1:** Genetic sequences used for phylogenetic reconstruction associated with their taxa, host, geographic origin, GenBank accession number and reference

Parasite taxa	Host	Geographic origin	GenBank	References
*Afrophilometra hydrocyoni*	*Hydrocynus forskahlii*	Kenya	JF803946	Moravec (2007)
*Alinema amazonicum*	*Callophysus macropterus*	Peru	DQ442672	Wijová et al. (2006)
*Anguillicola crassus*	*Anguilla anguilla*	Czech Republic	DQ490223	Wijová et al. (2006)
*Anguillicoloides australiensis*	*Anguilla australis*	Australia	JF805429	Laetsch et al. (2012)
*Anguillicoloides novaezelandiae*	*Anguilla australis*	Australia	JF805410	Laetsch et al. (2012)
*Anguillicoloides papernai*	*Anguilla mossambica*	Madagascar	JF805371	Laetsch et al. (2012)
*Camallanus lacustris*	*Sander lucioperca*	Czech Republic	DQ442663	Wijová et al. (2006)
*Dracunculus insignis*	*Procyon lotor*	USA	AY947719	Bimi et al. (2005)
*Dracunculus lutrae*	*Lutra lutra*	Canada	JF934737	Laetsch et al. (2012)
*Dracunculus medinensis*	*Homo sapiens*	Ghana	AY947720	Bimi et al. (2005)
*Dracunculus oesophageus*	*Natrix natrix*	Slovakia	AY852269	Wijová et al. (2006)
*Esocinema bohemicum*	*Esox lucius*	Russia	JF803917	Moravec (2007)
*Kalmanmolnaria intestinalis*	*Scardinius erythrophthalmus*	Russia	MH725826	Unpublished
*Kamegainema cingula*	*Andrias japonicas*	Japan	PV123245	Tsuchida et al. (2025)
*Mexiconema africanum*	*Auchenoglanis occidentalis*	Kenya	JF803947	Moravec (2007)
*Mexiconema cichlasomae*	*Cichlasoma urophthalmus*	Mexico	HM566089	Mejía-Madrid and Aguirre-Macedo (2011)
*Micropleura australiensis*	*Crocodylus johnsoni*	Australia	DQ442678	Wijová et al. (2006)
*Nilonema senticosum*	*Arapaima gigas*	Peru	DQ442671	Wijová et al. (2006)
*Philometra cyprinirutili*	*Abramis brama*	Czech Republic	DQ442675	Wijová et al. (2006)
*Philometroides sanguineus*	*Carassius carassius*	England	DQ442676	Wijová et al. (2006)
*Philonema oncorhynchi*	*Oncorhynchus kisutch*	Canada	DQ442670	Wijová et al. (2006)
*Philonema* sp.	Not informed	Not informed	U81574	Unpublished
*Rumai rumai*	*Arapaima gigas*	Brazil	JF803923	Moravec (2007)
*Sinoichthyonema amuri*	*Ctenopharyngodon idella*	Russia	MH725824	Sokolov et al. (2020)
*Skrjabillanus scardinii*	*Scardinius erythrophthalmus*	Russia	MH725825	Sokolov et al. (2020)
*Skrjabillanus tincae*	*Tinca tinca*	Russia	MH725827	Sokolov et al. (2020)

The phylogeny was reconstructed in the BEAST 2 software using Bayesian Inference (Bouckaert et al., 2019). The best-fit substitution model for the 18S alignment was chosen using the bModelTest package implemented in BEAST 2 (Bouckaert & Drummond, 2017), and the molecular clock model was relaxed-optimized defined using the nested sampling method (Russel et al., 2019). Parameter densities, ESS (estimated sample size), burn-in, and the chain convergence were examined in Tracer (Rambaut et al., 2018).

The posterior estimates of parameter densities, the ESS for each parameter, and the posterior probability for nodal supports in the majority rule consensus phylogenetic tree were, determined after running the Markov chain Monte Carlo (MCMC), using 4 chains in 2 runs for 10 × 10^6^ generations, with the sampling frequency at every thousand generation, with 25% burn-in, and saving the last 75% of generated trees.

## Results

### Systematics

Superfamily Dracunculoidea Stiles, 1907

Family Micropleuridae Baylis & Daubney, 1922

Genus *Micropleura* Linstow, 1906

Species *Micropleura vazi* Travassos, 1933

*Type host: Caiman crocodilus* (Linnaeus)

*Host of the present study: Caiman yacare* (Daudin)

*Site of infection:* Body cavity

*Geographic origins:* States of Rio de Janeiro and Mato Grosso in the original description by Travassos (1933a), and State of Mato Grosso do Sul (19°33′42′′S, 57°19′40′′W) in the present study, all from Brazil

*Voucher specimens:* 9 gravid females (CHIOC 39865)

*Intensity of infection:* 15 parasites in the single host analysed

#### Redescription (Figs. 1 and 2).

**Fig. 1:**
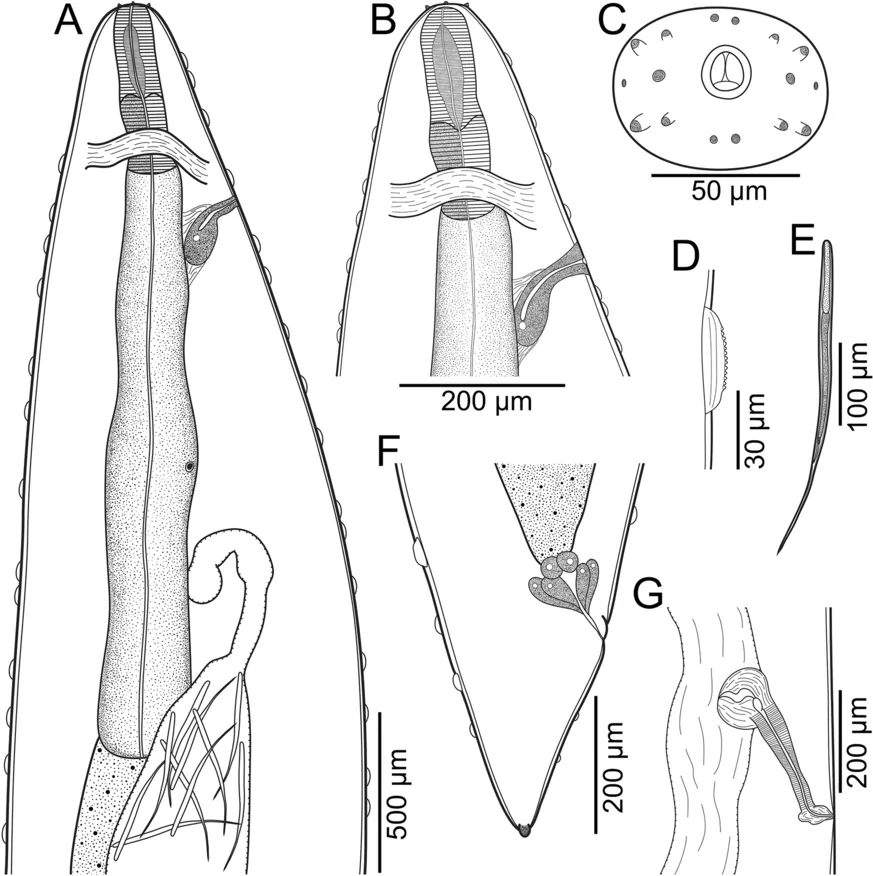
Female of *Micropleura vazi* Travassos, 1933 ex. *Caiman yacare* (Daudin). **A** and **B** Anterior end, lateral views. **C** Cephalic end, apical view. **D** Cuticular bosse lateral view. **E** First stage larva extracted from uterus, lateral virew. **F** Posterior end, lateral view. **G** Region of vulva, lateral view

**Fig. 2:**
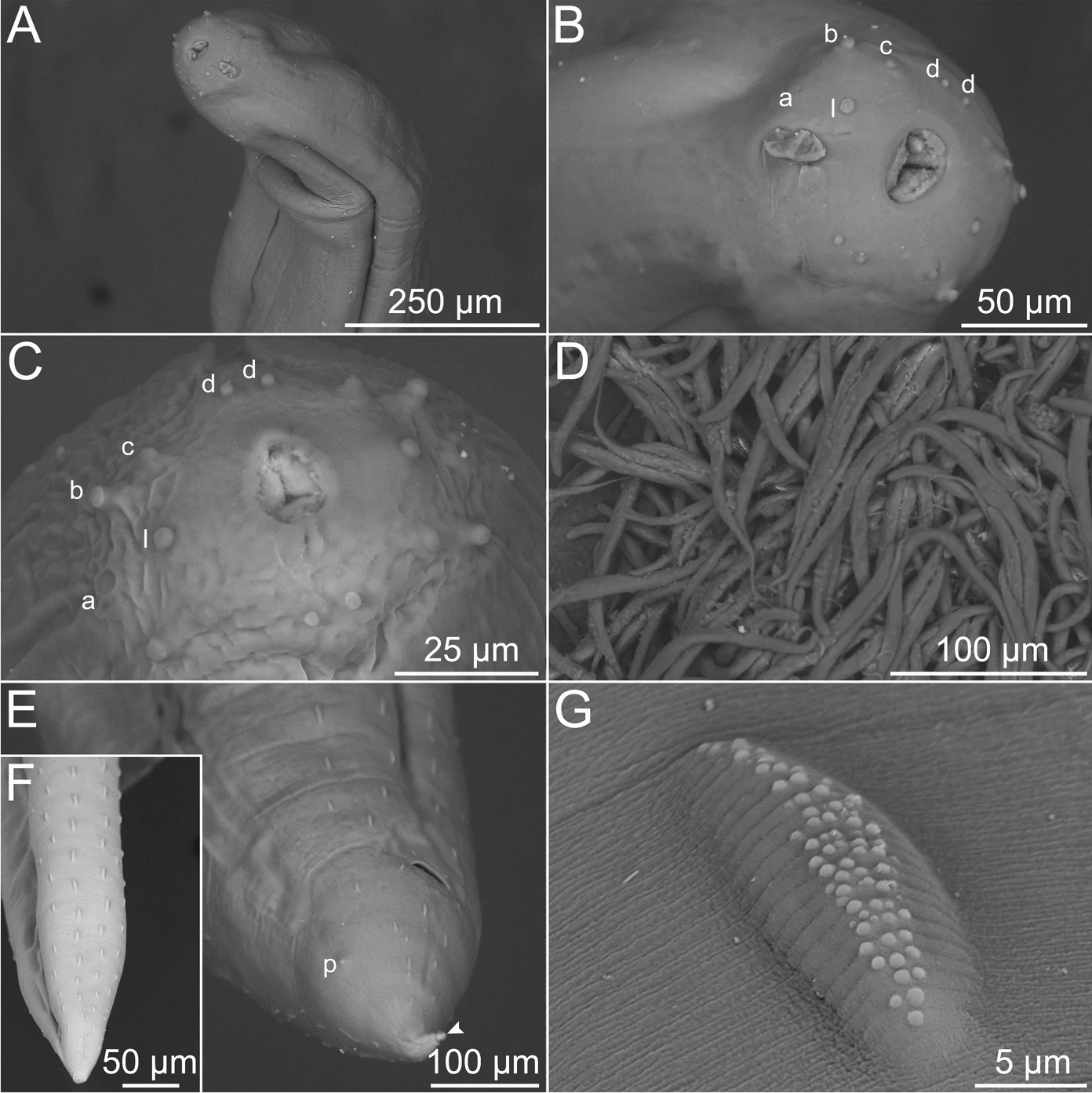
Scanning electron microscopy of females of *Micropleura vazi* Travassos, 1933 ex. *Caiman yacare* (Daudin). **A** Anterior end, apical view. **B** and **C** Cephalic end, apical views. **D** First stage larvae extracted from uterus. **E** and **F** Posterior end, sublateral and dorsal views, respectively (arrowhead indicates mucron). **G** Cuticular bosse, subapical view. Abbreviations: a, amphid; b, subdorsal/subventral cephalic papilla with pedunculate aspect; c, subdorsal/subventral smaller cephalic papilla; d, dorsal/ventral pair of cephalic papillae; l, lateral cephalic papilla; p, phasmid

Female (based on 10 gravid and 3 non-gravid specimens; measurements of non-gravid specimens inside parentheses): Long and robust nematodes, whitish opaque when alive and fixed, slightly tapered at both ends (Figs 1A and B, 2A, B, E, and F); 15.62–21.49 (8.2–10.2) mm long and 1.1–1.3 (0.586–0.718) mm wide. Cuticle thin, with smooth transverse striations (Fig. 2G) and numerous conspicuous cuticular protrusions (bosses) distributed throughout the body, ornamented with small, rounded structures (papilla-like) on their tops, about 34–51 long and 8–10 height (Figs. 1D and 2G). Anterior end dome-shaped, oral opening simple, oval, slightly depressed, surrounded by slightly elevated peribuccal ring, its bottom formed by surfaces of three oesophageal sectors resembling pseudolabia (Figs. 1C, 2B and C). Fourteen conspicuous cephalic papillae distributed as follows: four pairs with pedunculate aspect two subventral and two subdorsal, two smaller pairs one dorsal and one ventral, and one pair larger and lateral (Figs 1C, 2B, and C). Pore-like amphids located laterally to the lateral cephalic papillae (Figs 1C, 2B, and C). Deirids not observed. Buccal capsule absent (Fig. 1A and B). Oesophagus divided into shorter anterior muscular and longer posterior glandular portions (Fig 1A and B). Total length of oesophagus 2.2–2.6 (1.6–1.8) mm, representing 12.1–14.7 (17.6–21.2)% of total body length. Muscular oesophagus with complex musculature, 483–570 (295–399) long and 112–147 (75–94) wide (Fig 1A and B). Glandular oeosphagus superposed to muscular oesophagus on its dorsal part, 1.71–2.03 (1.3–1.5) mm long and 253–359 (185–229) wide, containing one mid-sized nucleus at about 1.42–1.48 (0.93–1.1) mm from anterior end (Fig 1Aa and B), sometimes hard to observe. Ratio of muscular to glandular oesophagus length 1:2.4–3.0 (1:2.2–2.6). Nerve ring slightly anterior to muscular-glandular oesophagus junction, 408–558 (232–317) from the anterior end (Fig 1A and B). Excretory pore associated to conspicuous excretory glandular cell, 382–741 (379–453) from the anterior end (Fig 1A and B). Vulva atrophied, flowed by short vagina with striated musculature, muscular ovijector anteriorly directed and globular sphincter with striated musculature, better visualized in non-gravid specimens (Fig. 1G), 8.06–10.23 (3.7–5.2) mm from the anterior end, at 35.7–47.6 (17.2–24.2)% of body length. Didelphic and amphidelphic uterus, saccular, occupying most part of body, anteriorly reaching posterior part of glandular oesophagus, full of first stage larvae in gravid females (Fig. 1A), and egg-shaped cellular formations in immature females. Ovaries distal, short, with rounded ends (Fig. 1A). Narrow rectum, surrounded by six well-developed cellular glands (Fig. 1F). Tail conical, 261–356 (203–224) long, with terminal mucron in gravid specimens (Fig. 1F) and without it in immature specimens.

First stage larva (based on five specimens extracted from uterus of one gravid female): Small and slender body, 356–405 long and 14–16 wide (Figs 1E and 2D). Oesophagus 83–93 long, representing 21.5–24.4% of body length. Tail long, conical and sharply pointed, 120–125 in length, representing 30.0–34.8% of body length (Figs 1E and 2D).

### Remarks

Since the present females have weakly developed buccal cavity (almost absent), internal cephalic papillae prominent (with peduncular aspect) and long glandular oesophagus, they were classified within the family Micropleuridae, which comprises only the genus *Micropleura*, represented by typical parasites of body cavity in crocodilians and chelonians (*sensu* Anderson et al., 2009). Notably, Anderson et al. (2009) state that member of Micropleuridae lack a peribuccal ring, yet this structure was observed in both the present specimens and those studied by Moravec & Prouza (2003).

Currently, the only species of *Micropleura* reported in the Americas, specifically within the Neotropical Region, is *M*. *vazi* which parasitizes caimans including *C*. *yacare* (see Moravec & Prouza, 2003). Although only females were found in the present study, their morphology closely matches that described by Moravec & Prouza (2003), particularly in the cephalic structures (number and arrangement of cephalic papillae and amphids; presence of peribuccal ring), the morphology and distribution of cuticular bosses throughout the body, and the aspects of the tail in both gravid (with mucron) and non-gravid (without it) specimens. Moreover, the morphology of the first stage larva was also the same among the present material and the previous descriptions of *M*. *vazi* (Travassos, 1933a; Moravec & Prouza, 2003). Based on this morphological evidence, and on the similar hosts and geographic origins (see Travassos, 1933a and Moravec & Prouza, 2003) we conclude that the present material belongs to the species *M*. *vazi*.

Gravid females of *M*. *vazi* have wide variation in their body length, ranging from 15.6 mm to 30.2 mm (Travassos, 1933a; Moravec & Prouza, 2003; present study). Interestingly, the morphometry of internal organs and structures, including the oesophagi, nerve ring and excretory pore locations, tail length, and certain proportions, is notably less variable and more conspicuous. The present morphometric data align with those provided by Moravec & Prouza (2003), except for the length ratio of muscular to glandular oesophagus. This minor discrepancy may be attributed to intrinsic characteristics of parasite and host populations, as well as environmental factors. Unfortunately, the original description of *M*. *vazi* by Travassos (1933a) did not provide detailed morphometric information, although the morphological description is considered complete in several aspects.

SEM observations are crucial for a better understanding of nematode parasite morphology, which is emphasized by Moravec et al. (2004) for visualizing cephalic papillae in *Micropleura* spp. This is well illustrated by the fact that Travassos (1933a), limited by the technology of the time, reported only three pairs of cephalic papillae in *M*. *vazi*. This finding was latter corrected by Moravec & Prouza (2003), who provided the first observations on the species using SEM. Although, the SEM micrographs provided by these authors were informative, they lacked clarity, particularly regarding the cephalic region. The current SEM micrographs offer improved visualization of this region and document the first stage larvae and the phasmid for the first time.

Oesophageal gland nuclei have been reported only in *M*. *australiensis*, which is a species morphologically similar to *M*. *vazi* (Moravec et al., 2004). This is the first time that these structures are described in *M*. *vazi*, suggesting that they may be more frequent in the genus than what is currently known. In fact, during our observations was possible to note the darkened aspect of the glandular oesophagus of *M*. *vazi*, making it difficult to observe the oesophageal gland nucleus.

Finaly, while Moravec & Prouza (2003) could not observe the vulva, Travassos (1933a) indicated that its position was median. Here, we found that the vulva is in fact in the anterior half of body. Since Travassos (1933a) did not perform detailed morphometric evaluation, the vulval location could have been reported imprecisely on these large female nematodes. Moreover, we also observed for the first time that both vagina and ovijector are anteriorly directed in *M*. *vazi*, differing it from *M*. *australiensis* and *M*. *huchzermeyeri* that have accurate descriptions for these characters, in which they direct posteriorly (Moravec et al., 2004; Junker & Mutafchiev, 2017).

### Genetic characterization and phylogeny

We obtained partial fragments of the genes 18S (887 bp) and 28S (367 bp) rDNA. The following analyses were based solely on 18S sequences, due to the data availability for dracunculoids and, especially, for species of *Micropleura*. A preliminary BLAST search indicated high genetic similarity between the present sample and different isolates of the congener *M*. *australiensis* (96.6–96.7%).

**Fig. 3:**
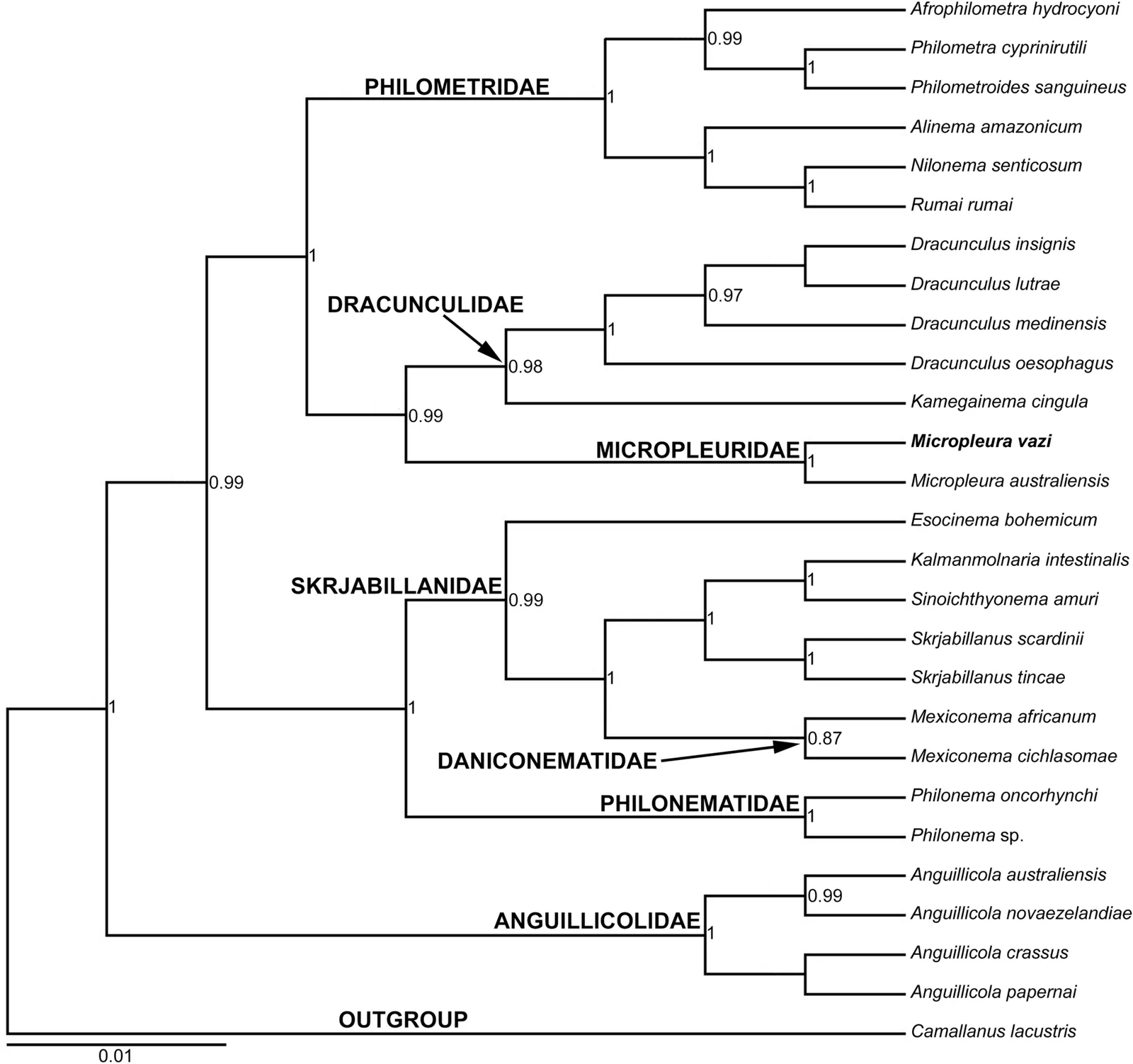
Phylogenetic reconstruction using Bayesian inference from 18S rDNA sequences (model TN93 + I + G) of dracunculoid (Dracunculoidea) nematodes. Family names are associated with each clade. Nodal support is given as Bayesian posterior probabilities after running the Markov chain Monte Carlo (2 runs, 4 chains, 1 × 10^7^ generations, 25% burn in) and values under 0.85 are omitted. The representative of the present work is in bold

The phylogeny was reconstructed based on the alignment of 18S sequences (877 sites), representing the 5′ end of the gene, and the substitution model was the TN93 + I + G, assuming unequal nucleotide frequencies. The genus *Micropleura* and consequently the family Micropleuridae, represented by only *M*. *australiensis* and *M*. *vazi*, were monophyletic (Fig. 3). These taxa were sister to Dracunculidae (Fig. 3). All families within Dracunculoidea were monophyletic, except Skrjabinellanidae, which was paraphyletic, and the most basal was Anguillicolidae (Fig. 3). All these phylogenetic assemblages, except for that of Daniconematidae, showed high to full support (Fig. 3).

## Discussion

The taxonomy of *Micropleura* remains challenging due to the insufficient morphological data for many species. For instance, Moravec et al. (2004) synonymized *M*. *trionyxi* Agrawal, 1966 and *M. lissemysia* Chattervati, 1985 with *M*. *indica*, all parasites of chelonians from India, because of the poorly detailed descriptions regarding the first two. Moreover, *M. helicospicula* Dey Sarkar, 2003, described from *Cr*. *palustris* Lesson in India (Dey Sarkar, 2003), remains of uncertain taxonomic status and an incomplete morphological description. Regarding *M*. *vazi*, its cephalic morphology was ambiguous until SEM observations were performed on the species (see Travassos, 1933a; Moravec & Prouza, 2003). The complex taxonomic situation is further exacerbated by the lack of genetic data on these nematodes. Therefore, comprehensive morphological assessments and genetic analyses are important to improve the taxonomic understanding on the genus *Micropleura*, as the data provided here.

Even though the morphological knowledge on *Micropleura* spp. can be considered generally incipient, significant advances have occurred over the past 20 years, mainly due to the studies by Moravec & Prouza (2003), Moravec et al. (2004, 2006) and Junker & Mutafchiev (2017), which provide detailed descriptions and SEM observations. The same cannot be said for the molecular data, in which only *M*. *australiensis* has been genetically characterized to date. This limitation restricts the comprehensive phylogenetic analysis of *Micropleura*. Nevertheless, the inclusion of the 18S sequence of *M*. *vazi* supports the continued recognition of both Micropleuridae and *Micropleura* as valid and monophyletic group.

Molecular phylogenetic evidence strongly supports the monophyly of Dracunculoidea and its position as sister group of Camallanoidea (see Wijová et al., 2006; Nadler et al., 2007; Černotíková et al., 2011; Sokolov et al., 2020). However, since Dracunculoidea is a highly diverse superfamily including families diverging among distantly related groups of vertebrates (mainly fish, reptiles and mammals) (Anderson et al., 2009), its systematics remain debated. Recent phylogenetic advances may help resolve these systematic uncertainties, which will be discussed as follows.

The present results reinforce the valid status of Micropleuridae as an independent family, distinct from Philometridae, different from the classification proposed by Travassos (1933a) that included *Micropleura* within the former. Similarly, *Philonema* Kuiten-Ekbaum, 1933 has been classified within Philometridae (Nadler et al., 2007; van Megen et al., 2009; May-Tec et al., 2019) or Micropleuridae (Moravec, 2006). However, the current results demonstrate that *Philonema* is clearly separated from representatives of both families. In this sense, we agree with the proposal of Sokolov et al. (2020) in that the family Philonematidae should be considered valid, at least provisionally.

The phylogenetic position of Micropleuridae remains unresolved, although it is closely related to Dacunculidae and Philometridae (Wijová et al., 2006; Černotíková et al., 2011; Sokolov et al., 2020). In the present analysis, Micropleuridae was the sister group of Dracunculidae forming a clade sister to Philometridae. This arrangement differs slightly from the results by Sokolov et al. (2020). These discrepancies likely result from differences in the dataset used for phylogenetic reconstruction; in which Sokolov et al. (2020) did not include the genus *Kamegainema* Hasegawa, Doi, Araki & Myiata, 2000 (Dracunculidae) parasitic in amphibians, due to the absence of data at the time. Moreover, methodological differences may have contributed, as the support of Bayesian inference for their phylogeny was low. The results of Tsuchida et al. (2025), which included *Kamegainema* in their analysis, corroborate the present findings by yielding similar Bayesian phylogenetic results to those of the present study. Overall, the evidence suggests that Micropleuridae is ancestral to Dracunculidae as previously indicated by Chabaud & Bain (1994). This inference is further supported by the narrower host spectrum of Micropleuridae (crocodilians and turtles) compared to the broader host range of Dracunculoidea (amphibians, reptiles, birds and mammal), which aligns with the ancestral and derived characteristics described by these authors.

The genus *Kamegainema* was retained in Micropleuridae by Gibbons (2010). However, *Kamegainema* seems to be more closely related to *Dracunculus* than to *Micropleura* supporting its placement within Dracunculidae (see also Moravec, 2006; Tsuchida et al., 2025). In fact, the inclusion and exclusion of genera within Micropleuridae have been complicated, and some authors currently include *Protenema* Petter & Planelles, 1986 and *Granulinema* Moravec & Little, 1988 in this family (for more detail see Moravec 2006; Gibbons, 2010). Unfortunately, there is no representative of Micropleuridae other than *Micropleura* with molecular data for phylogenetic analysis. Therefore, the classification of Anderson et al. (2009) is followed here, considering Micropleuridae with the single genus *Micropleura*.

Similarly, the family Daniconematidae which comprises two genera in addition to *Mexiconema* Moravec, Vidal & Slagado-Maldonado, 1992 namely, *Daniconema* Moravec & Køie, 1987 and *Syngnathinema* Moravec, Spangenberg & Frasca, 2001 (Gibbons, 2010), seems to be artificial according to the results of May-Tec et al. (2019). The inclusion of Daniconematidae rendered Skrjabillanidae paraphyletic in the present analysis; this lack of monophyly has been also observed in other studies (Černotíková et al., 2011; Sokolov et al., 2020). Additional phylogenetic data are needed to determine whether Daniconematidae should be considered synonymous with Skrjabillanidae, as the type genus of the former (*Daniconema*) has not yet been genetically characterized.

Another focus of discussion relies on the Anguillicolidae Yamaguti, 1935, a family of ichthyoparasites endemic to freshwater eels of the genus *Anguilla* (Laetsch et al., 2012). This family has been historically placed into Dracunculoidea (Chabaud & Bain, 1994; Wijová et al., 2006; Anderson et al., 2009; Gibbons, 2010), but more recently has been considered in a separated superfamily namely Anguillicoloidea (Moravec, 2006; Laetsch et al., 2012; Sokolov & Gordeev, 2021). According to previous phylogenetic results (see Wijová et al., 2006; Laetsch et al., 2012; Sokolov & Gordeev, 2021) the anguillicolids (or anguillicoloids) are far from dracunculoids and closer to Gnathostomatoidea, although their exact phylogenetic position among the Spirurina (*sensu* Wijová et al., 2006; Laetsch et al., 2012) remains not fully clear. Our results showed that Anguillicolidae was basal and sister to the other dracunculoids, acting as an outgroup. In fact, if *Camallanus lacustris* (Zoega, 1776) is not constrained as the outgroup, this function is be assumed by the Anguillicolidae and *C*. *lacustris* would appear as sister to the drancunculoids. Accordingly, we agree with the previous proposals regarding the validity of Anguillicoloidea (Moravec, 2006; Laetsch et al., 2012) and that these nematodes are not closely related to the Drancunculoidea (Wijová et al., 2006). Morphological and biological evidence further support this distinction (see Moravec, 2006; Wijová et al., 2006). It seems that Anguillicoloidea holds several ancestral features in relation to Dracunculoidea (see Wijová et al., 2006; Ailán-Choke et al., 2023).

Here, we provide detailed morphological observations on the females of *M*. *vazi*, making it possible to note previously unreported features and to elucidate some inconsistencies. The absence of males in our sampling may be related to the biology of the parasite. Although the life cycle of *Micropleura* spp. remains practically unknown, the occurrence of males is probably seasonal, as has been reported for other dracunculoid nematodes (Anderson, 2000; Moravec, 2004). Moreover, during the necropsy we analysed the whole carcass of the single *C*. *yacare*, since it is also known that dracunculoid males and females may occupy different sites of infection (Anderson, 2000; Moravec, 2004), but we found no male.

The data provided here strengthens the specific diagnosis of *M*. *vazi*, and the new genetic sequence helped a better understanding of the phylogenetic relationships between Micropleuridae and other dracunculoid nematodes. The phylogenetic analysis focused on the Dracunculoidea also assisted in the evaluation of some systematic debates and confirmation of some previous classification proposals. Still, much needs to be done for us to truly understand the evolutionary relationships between these interesting parasites.

## Data Availability

All data is available in the manuscript or deposited in public repositories (biological collection and GenBank).
